# Solid Electrolyte Interphase and Interface Effect on the Nucleation of Lithium Pitting

**DOI:** 10.1021/acsnano.5c16454

**Published:** 2026-01-17

**Authors:** Hanrui Zhang, Weixi Tian, Yanjun Guo, Dongliang Chen, Feifei Shi

**Affiliations:** † John and Willie Leone Family Department of Energy and Mineral Engineering, 311285 The Pennsylvania State University, University Park, Pennsylvania 16802, United States; ‡ Department of Materials Science and Engineering, The Pennsylvania State University, University Park, Pennsylvania 16802, United States

**Keywords:** nucleation of lithium pitting, charge transfer (CT), solid electrolyte interphase (SEI), 2D nucleation, 3D nucleation

## Abstract

The nucleation of pits and their evolution on the lithium (Li) metal anode greatly impact the cyclability and safety of Li–metal batteries. Overpotential has been found to be inversely related to the Li pit radius and exponentially related to the nucleation rate. However, it remains unclear how the electrode/electrolyte interphase and interface impact the nucleation of pitting. In this study, we decouple the electrolyte effect into a solid electrolyte interphase (SEI) and interface charge transfer (CT). Under galvanostatic stripping, ether electrolytes yield larger and sparser pits than the carbonate electrolyte, which is related to the lower stripping overpotential of ether electrolytes. Under potentiostatic stripping, a smaller pit size and higher nucleation density have been revealed in ether electrolytes due to the higher nucleation rate. We found that charge-transfer kinetics on the interface of the Li-metal anode will greatly influence its nucleation mode and kinetics. Slow charge-transfer kinetics in the carbonate electrolyte lead to a lower nucleation rate and larger Li pits, which are close to 3D nucleation. In ether electrolytes with fast charge transfer kinetics, the pitting process exhibits 2D nucleation. The SEI derived from various electrolytes mainly influences the stripping overpotential and the morphology of the pits. By clarifying the roles of SEI and interfacial charge transfer on the nucleation of pitting, this work will greatly benefit the design of cycling profiles and electrolyte recipes for next-generation Li-metal batteries.

## Graphical abstract

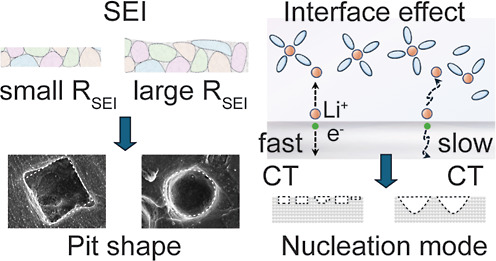

## Introduction

Rechargeable lithium-metal batteries represent a promising path to next-generation energy storage with high energy density.^1,2^ However, the Li-metal anode undergoes significant volume expansion and nonuniform morphological changes during the deposition/stripping process.^3,4^ The nucleation processes of deposition and stripping greatly impact the morphology of the Li anode electrode during cycling.^5,6^ Compared to the process of metal deposition nucleation, the nucleation mechanisms of the stripping/pitting reaction are less known or explored.^7^ Our group’s previous study validated that the overpotential is inversely related to the pit radius and exponentially related to the rate of nucleation.^8^ This finding highlights the critical role of overpotential in governing pit nucleation, yet the electrolyte effect on the nucleation of pitting has been barely explored. Most stripping studies focused on single-recipe electrolytes, such as carbonate-based electrolytes.^9–11^ The effect of electrolyte on the Li-metal anode is usually composed of multiple coupled factors, like solid electrolyte interphase (SEI), interface charge transfer, and desolvation process, which makes it challenging to clarify the primary origin of the pitting nucleation.^12,13^

According to classic nucleation theory (CNT), the nucleation mode can be classified into 2D nucleation and 3D nucleation.^14^ 2D nucleation proceeds layer by layer, producing relatively smooth and uniform surfaces, while 3D nucleation initiates as discrete islands, leading to a rougher surface.^15^ Gretz et al. found that nucleation is determined by adsorption and charge transfer across the surface in chemical vapor deposition (CVD).^16^ Kajikawa and Noda reported that in physical vapor deposition (PVD) and CVD processes, the thin film typically follows a 3D nucleation. By enhancing the reaction kinetics, such as surface energy and substrate reactivity, the nucleation of thin film (such as Si and Cu) will transition to a 2D mode.^17^ In terms of Li deposition, Thirumalraj et al. reported that Li undergoes a 3D nucleation mode under potentiostatic deposition and proposed a Li–SEI nucleation model with the electrolyte as 1 M in LiPF_6_ EC/DEC.^18^ Despite these advances, the nucleation mode of Li pitting has never been investigated. Understanding this process is critical for controlling the morphology of the Li anode and designing the cycling profiles.

In this work, we studied the pitting behavior of the Li-metal anode in 3 commonly used electrolytes, namely, 1 M LiPF_6_ in EC/DEC (LP40), 1 M LiTFSI in DOL/DME with 1 wt % LiNO_3_ (D/D), and LiFSI/DME/TTE = 1:1.2:3 by mol (LHCE-M47). Combining optical microscopy (OM) with confocal laser scanning microscopy (CLSM), we systematically examined the evolution of pit size and morphology in both 2D and 3D views. Statistical analysis of large data sets (>500 pits per condition) allowed us to correlate the nucleation mode (2D vs 3D) of Li pitting under galvanostatic stripping. Under potentiostatic stripping in a 3-electrode cell, mathematical fitting of the *i*–*t* curves revealed that Li pitting follows 2D instantaneous nucleation in ether-based electrolytes, whereas carbonate-based electrolytes favor 3D instantaneous nucleation. Furthermore, time-dependent electrochemical impedance spectroscopy (EIS) combined with Raman and FT-IR spectroscopy was employed to probe the desolvation, SEI resistance (*R*_SEI_), and charge-transfer resistance (*R*_CT_) across 3 electrolytes. The results show that the SEI regulates the overpotential of stripping and the morphology of Li pits. Rapid interfacial charge transfer favors 2D instantaneous nucleation, whereas sluggish charge transfer leads to the 3D instantaneous nucleation of pitting. This work provides new insights into how the SEI and the interface govern the early-stage nucleation and growth of Li pitting.

## Results and Discussion

### Pitting of Li under Galvanostatic Stripping

The previous studies on Li pitting focused on the carbonate-based electrolytes.^9–11^ In this work, we use 3 widely applied electrolytes (LP40, D/D, and LHCE-M47) to investigate the electrolyte effect on the nucleation and evolution of Li pits, and the details about electrolyte selection are discussed in Supporting Information Note 1. We stripped Li-metal anodes in 3 electrolytes at various current densities with a fixed areal capacity of 0.2 mAh cm^–2^. Over 1000 pits are counted for each current density, and the pit morphology and size distribution of Li pits in LHCE-M47 are shown in Figure 1a. As shown in Figure 1a, in the LHCE-M47 electrolyte, the median pit radius is 14.30, 12.22, 12.06, 11.23, 9.76, and 8.57 μm from 0.5–5 mA cm^–2^. In the D/D electrolyte (Figure S1), the median pit radius is 13.34, 12.08, 11.17, 10.69, 10.23, and 7.99 μm when stripped under 0.5–5 mA cm^–2^, which is slightly smaller than that of LHCE-M47. The zoom-out images can be found in Figures S2 and S3 for LHCE-M47 and D/D. Figure 1b summarizes the pit size of Li stripped in 3 electrolytes, where the LP40 data is retrieved from our previous work.^8^ With the same galvanostatic stripping conditions, ether-based electrolytes (D/D, LHCE-M47) will generate larger pits than the carbonate-based electrolyte (LP40). This trend is due to the smaller overpotential under the same stripping current density, as shown in Figure S4. The ionic conductivity of 3 electrolytes is very close and lies in the range of 10^–3^ to 10^–2^ S cm^–1^, as exhibited in Figure S5. Other than ionic conductivity, their other physical properties, such as viscosity and density, are also very close.^19^ Therefore, the observed differences originate from interfacial effects, such as Li^+^ transport across the SEI and charge-transfer kinetics, rather than from bulk electrolyte physical properties.

**Figure 1:**
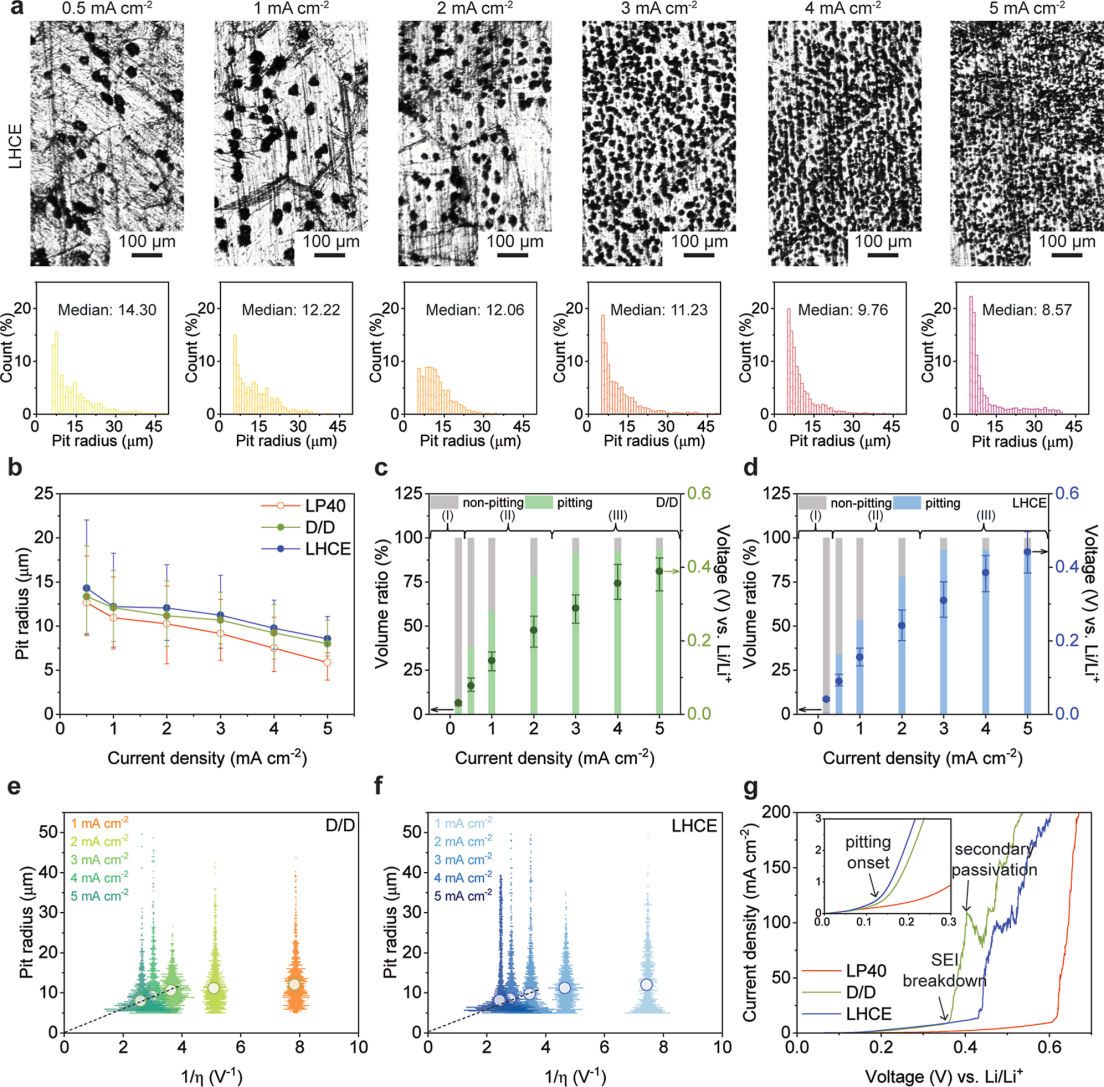
Pit size distribution under galvanostatic stripping. (a) The morphology and pit size distribution of Li after stripping in the localized high-concentration electrolyte (LHCE-M47, LiFSI/DME/TTE = 1:1.2:3 by mol). The stripping current densities are 0.5, 1, 2, 3, 4, and 5 mA cm^–2^, respectively. The stripping capacity is fixed at 0.2 mAh cm^–2^. (b) The pit radius in 3 electrolytes: LP40 (1 M LiPF_6_ in EC/DEC 1:1 vol %), D/D (1 M LiTFSI in DOL/DME 1:1 vol % with 1 wt % LiNO_3_), and LHCE-M47. Median values are reported, and error bars represent the 20%–80% percentile range. The volume ratio of pits in (c) D/D and (d) LHCE-M47 electrolytes. The total stripped volume of Li is calculated based on the theoretical capacity, the overpotential is extracted with 3-electrode setup. Correlation between the pit radius and the inverse of the overpotential in (e) D/D and (f) LHCE-M47 under different stripping current densities. (g) The anodic polarization curves for Li foil with SEI formed in different electrolytes. The linear sweep voltammetry is conducted with a 3-electrode Li/Cu cell, with LTO as the reference electrode. The scan rate is 0.1 mV s^–1^. Li-metal anodes are aged for 24 h to form a stable SEI.

In our previous work, we identified three stripping modes for the Li anode, namely, stripping without pitting (I), mixed mode (II), and stripping with mainly pitting (III).^8^ Assisted by a CLSM, we quantify the total volume stripped through pitting and identify the stripping mode in the D/D and LHCE-M47 system. In the D/D electrolyte (Figure 1c), there is negligible volume (<10%) stripped through pitting when the current is 0.2 mA cm^–2^. This region is under stripping without the pitting mode (I). When currents increase, the pitting volume also increases to 38.2%, 58.7%, and 78.4% at 0.5, 1, and 2 mA cm^–2^, respectively. It follows a mixed stripping mode where pitting and nonpitting both contribute to the stripped volume. When the current density exceeds 2 mA cm^–2^, the pitting is the dominant mode for stripping with mode I occurring at current density <0.2 mA cm^–2^, mode II between 0.5–2 mA cm^–2^, and mode III at currents >2 mA cm^–2^. In the LP40 electrolyte, the mode III is triggered when the current density exceeds 1 mA cm^–2^,^8^ whereas in D/D and LHCE-M47 electrolytes, this transition does not occur until the current density reaches 2 mA cm^–2^.

In CNT, the driving force of electrochemical nucleation is the overpotential^14^

1 $$r_{c}=\frac{\gamma }{ne\eta }V_{a}$$

where γ is the interfacial energy, *n* is the number of electrons, *e* is the elementary charge of the electron, *V*_a_ is the atomic volume of Li, and η is the electrochemical overpotential.

The inverse of the pit radius as a function of overpotential in D/D and LHCE-M47 is shown in Figures 1e,f. A well-defined linear relationship is observed in the high-overpotential region, corresponding to the pitting-dominated mode (mode III). However, deviations from linearity occur in the mid-overpotential to low-overpotential regions due to the presence of mixed stripping modes or stripping without pitting. This trend is consistent across all 3 electrolytes, indicating that overpotential serves as the driving force for pitting nucleation, regardless of the electrolyte. These results indicate that the onset of different modes is similar across the 3 electrolytes. However, the smaller interfacial resistance in ether-based electrolytes requires higher current densities to reach the critical overpotential.

By using a 3-electrode Swagelok cell, we measured the anodic polarization curves of the Li-metal anode at a scan rate of 0.1 mV s^–1^. As shown in Figure 1g, the onset potential of pitting is approximately 0.1 V vs Li/Li^+^ for both carbonate and ether electrolytes. As the sweeping voltage increases, the current rises rapidly due to the pitting process. When the voltage reaches 0.35 V for D/D, 0.43 V for LHCE-M47, and 0.62 V for LP40, a sudden rise in current is observed, corresponding to SEI breakdown and the loss of interfacial passivity.^20^ For D/D reaching 0.4 V and LHCE-M47 reaching 0.45 V, a small potential plateau appears where the current barely changes with increasing voltage. This behavior corresponds to the “secondary passivation”, which is commonly observed in metal corrosion, signifying the formation of a new protective passivation layer that suppresses metal dissolution.^21^ As the voltage further increases, the newly formed passivation layer undergoes a second breakdown, leading to a sudden rise in current. Based on the critical transition voltages of the SEI breakdown, the SEI formed in the LP40 electrolyte has better passivity, which does not break down below 0.62 V.

Carbonate-based electrolytes typically yield SEI rich in Li_2_CO_3_ and organic polymeric species (e.g., ROCO_2_Li), which are thick and porous.^22^ The SEI formed in LHCE-M47 contains more inorganic species such as LiF,^23^ while the SEI in D/D has more Li_3_N.^24^ The SEI formed in the carbonate-based electrolyte is generally thicker than that formed in the ether-based electrolyte.^25^ The SEI formed in the carbonate electrolyte exhibits a higher breakdown voltage, which is mainly due to the large thickness. The SEI formed in LHCE-M47 shows a higher breakdown voltage than that formed in D/D due to higher LiF content.^26^ With a stripping current density less than 4 mA cm^–2^ (as shown in Figure S4 below), the stripping overpotential in the ether electrolyte is below the SEI breakdown voltage (0.35–0.43 V vs Li/Li^+^). In a full cell test with a cathode loading of 4 mAh cm^–2^, *C*/2 charging will generate only 2 mA cm^–2^ stripping current on the Li-metal anode side. Without a concern of breakdown, ether-based electrolytes will generate a better battery performance due to the lower internal resistance.

### The Incubation and Growth of Li Pits under Galvanostatic Stripping

To comprehensively understand the evolution of the pitting, the pits are examined with various stripping capacities from 0.2 mAh cm^–2^ to 2 mAh cm^–2^ at a fixed current density of 1 mA cm^–2^. As shown in Figure S6, the OM images show that the pits expand with increasing stripping capacity, and these pits formed in ether-based electrolytes are more faceted than those in carbonate-based electrolytes. The circularity distribution is shown in Figure S7 with over 500 pits counted for each condition as the measurement of circularity can be found in our previous work.^8^ The circularity evolution of pits under increasing capacities is shown in Figure 2a. For the LP40 electrolyte, the pit circularities are 0.815, 0.820, 0.809, and 0.816 under 0.2, 0.5, 1, and 2 mAh cm^–2^, respectively. This means the shape of the pits stripped in LP40 barely changes under the increasing capacity, indicating an isotropic growth mode. For D/D, the median circularity is 0.700, 0.6896, 0.6678, and 0.6448 from 0.2–2 mAh cm^–2^. LHCE-M47 shows a similar decreasing trend as D/D, with the median circularity as 0.723, 0.686, 0.674, and 0.6312 under the stripping capacities of 0.2, 0.5, 1, and 2 mAh cm^–2^, respectively. The decreasing circularity indicates that the growth mode of pits is anisotropic in D/D and LHCE-M47 electrolytes, where pits grow preferentially along specific crystal planes. Figure 2b shows the typical morphology of pits formed in 3 electrolytes at the capacity of 0.5 mAh cm^–2^. In OM and scanning electron microscopy (SEM) images, the pits formed in LP40 are of a rounded shape, while those formed in D/D and LHCE-M47 are more faceted in the in-plane direction.

**Figure 2:**
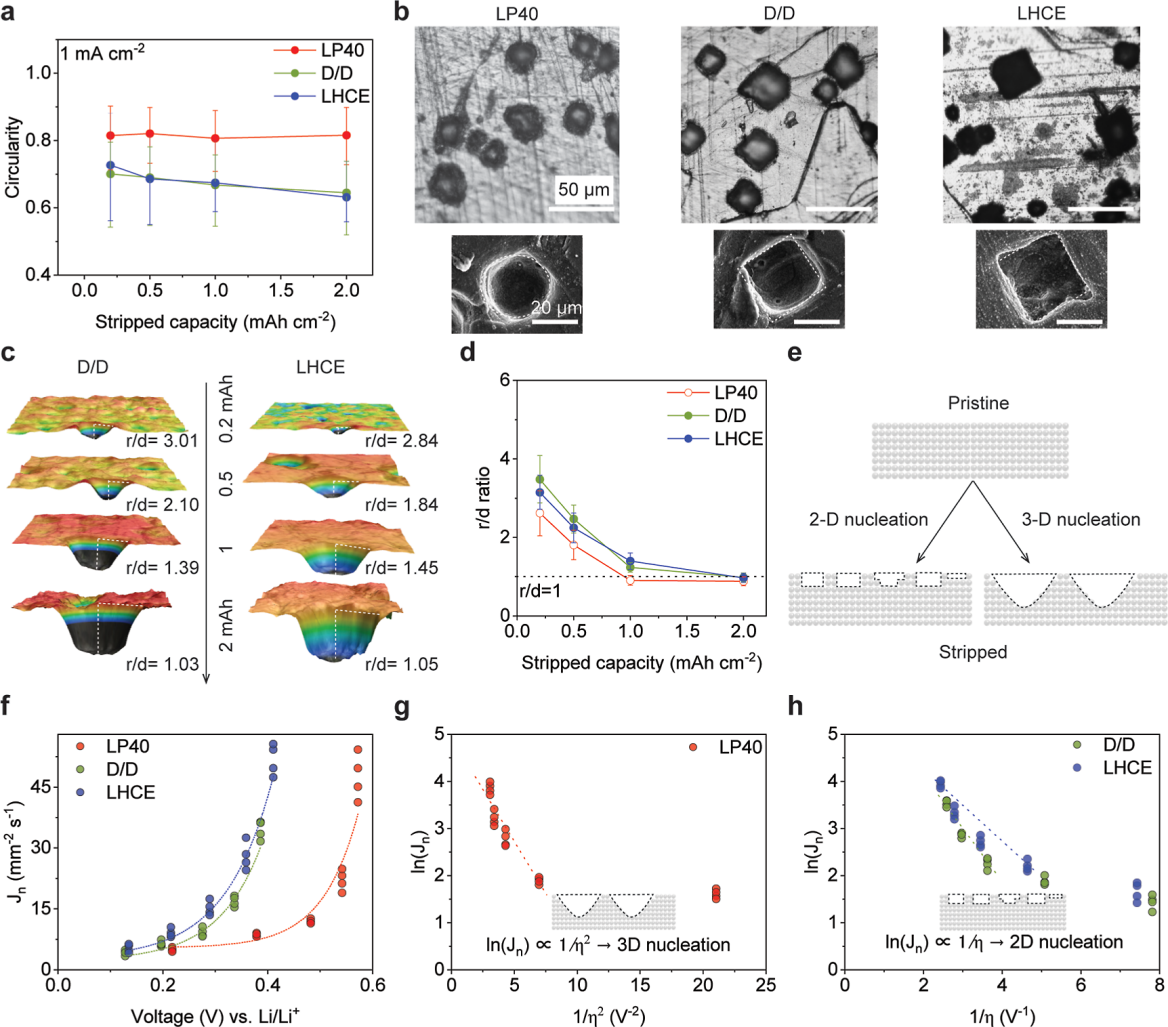
The incubation and growth of Li pits under galvanostatic stripping. (a) The circularity of Li pitting in 3 electrolytes with increasing stripped capacity. Median values are reported, and error bars represent the 20%–80% percentile range. (b) The typical morphology of Li pits formed in LP40, D/D, and LHCE-M47 electrolytes with a capacity of 0.5 mAh cm^–2^. (c) Cross sections of typical pit and *r*/*d* ratio of Li pits in D/D and LHCE-M47 electrolytes. (d) Evolution of the ratio between the pit radius and depth (*r*/*d*) of the stripped pits under various electrolytes and capacities, the stripping current is 1 mA cm^–2^, and over 500 pits are counted. Median values are reported, and error bars represent the 20%–80% percentile range. (e) The schematic of 2D nucleation and 3D nucleation of pitting. (f) The correlation between nucleation rate (*J*_n_) and nucleation overpotential (η). The correlation of the (g) natural logarithm of nucleation rate (*J*_n_) and the inverse of overpotential square (1/η^2^) and (h) natural logarithm of nucleation rate (*J*_n_) and the inverse of overpotential (1/η).

To quantify the evolution of pit growth at different directions (e.g., in-plane and through-plane), we use parameter *r*/*d*, which is defined as the aspect ratio of the pit radius (*r*) to the maximum pit depth (*d*). For a perfectly hemispherical pit, *r*/*d* equals 1. We utilized CLSM to obtain information from both in-plane and through-plane directions for *r*/*d* quantification. Figure 2c exhibits the cross-section of Li pits formed in the D/D and LHCE-M47 structures with different stripping capacities. With the increasing stripping capacities, the pits are initially wide and shallow, gradually evolving into a hemisphere-like shape to minimize the surface energy. The evolution of the ratio of *r*/*d* under increasing striped capacities is shown in Figure 2d. In the D/D electrolyte, the *r*/*d* values are 3.49, 2.46, 1.23, and 0.98 for current densities of 0.2, 0.5, 1, and 2 mAh cm^–2^, respectively. Similarly, in the LHCE-M47 electrolyte, the corresponding R values are 3.15, 2.24, 1.39, and 0.97. The *r*/*d*-value exhibits a consistent trend across the 3 electrolytes: it starts significantly greater than 1 and gradually approaches 1 as the stripping capacity exceeds 0.5 mAh cm^–2^.

At the early stage of stripping (low stripping capacity), pits in D/D and LHCE-M47 have a larger *r*/*d* ratio than those in LP40, indicating that they are shallower pits. This can come from the different nucleation mode. In the CNT, nucleation can be classified as either 2D or 3D. 2D and 3D nucleation modes govern the dimensions and the shape of the nuclei. In 2D nucleation, nuclei typically form as circular or elliptical monolayer islands on the surface. In contrast, 3D nucleation tends to evolve into a hemispherical or spherical shape to minimize interfacial energy.^27,28^ The schematics of 2D and 3D nucleation are shown in Figure 2e. In CNT, the governing equations for 3-D nucleation and 2D nucleation are

2 $$\ln\left(J_{n}\right)\propto \left(-\frac{1}{\eta^{2}}\right)\left(3D\;nucleation\right)$$

3 $$\ln\left(J_{n}\right)\propto \left(-\frac{1}{\eta }\right)\left(2D\;nucleation\right)$$

where *J*_n_ is the nucleation rate and η is the overpotential.^14^ The detailed derivation can be found in Note S2. With the constant current stripping mode, the nucleation time varied under different conditions. The determination of nucleation time has been reported in our previous work.^8^ Figure 2f shows the correlation between *J*_n_ and nucleation overpotential η. It shows that the pitting nucleation follows an exponential relationship in all 3 electrolytes. We further explore the relationship among the natural logarithm of nucleation rate ln­(*J*_n_), inverse of overpotential square 1/η^2^ (Figure 2g), and inverse of overpotential 1/η (Figure 2h). For the LP40 electrolyte, ln­(*J*_n_) shows a linear dependence on 1/η^2^ at large overpotentials, indicating a 3D nucleation mode. In contrast, ln­(*J*_n_) exhibits a linear dependence on 1/η for both D/D and LHCE-M47 electrolytes. However, the linear correlation remains weak, which can be attributed to the dynamic nucleation potential under galvanostatic stripping. With a variable overpotential of nucleation, it is challenging to clarify the nucleation rate and nucleation mode of Li pitting.

### Static Nucleation of Li Pitting under Potentiostatic Stripping

To eliminate dynamic overpotential η with a galvanostatic approach, we switch to a 3-electrode potentiostatic setup for the study of steady-state nucleation. The current and voltage profiles of galvanostatic and potentiostatic approaches are compared in Figure 3a,b. Under the potentiostatic measurement, the current–time transients can be normalized with respect to the peak current (*I*_M_) and its corresponding time (*t*_M_), and subsequently compared to 4 classical nucleation models: 3DI, 3DP, 2DI, and 2DP. “I” denotes instantaneous and “P” denotes progressive nucleation. The Scharifker–Hills models (3DI and 3DP) describe planar diffusion-controlled three-dimensional growth of hemispherical nuclei.^29^ In contrast, the Bewick–Fleischman–Thirsk models (2DI and 2DP) represent two-dimensional lateral growth of cylindrical nuclei.^30^ The mathematical expressions of these models are provided in Note S3.

**Figure 3:**
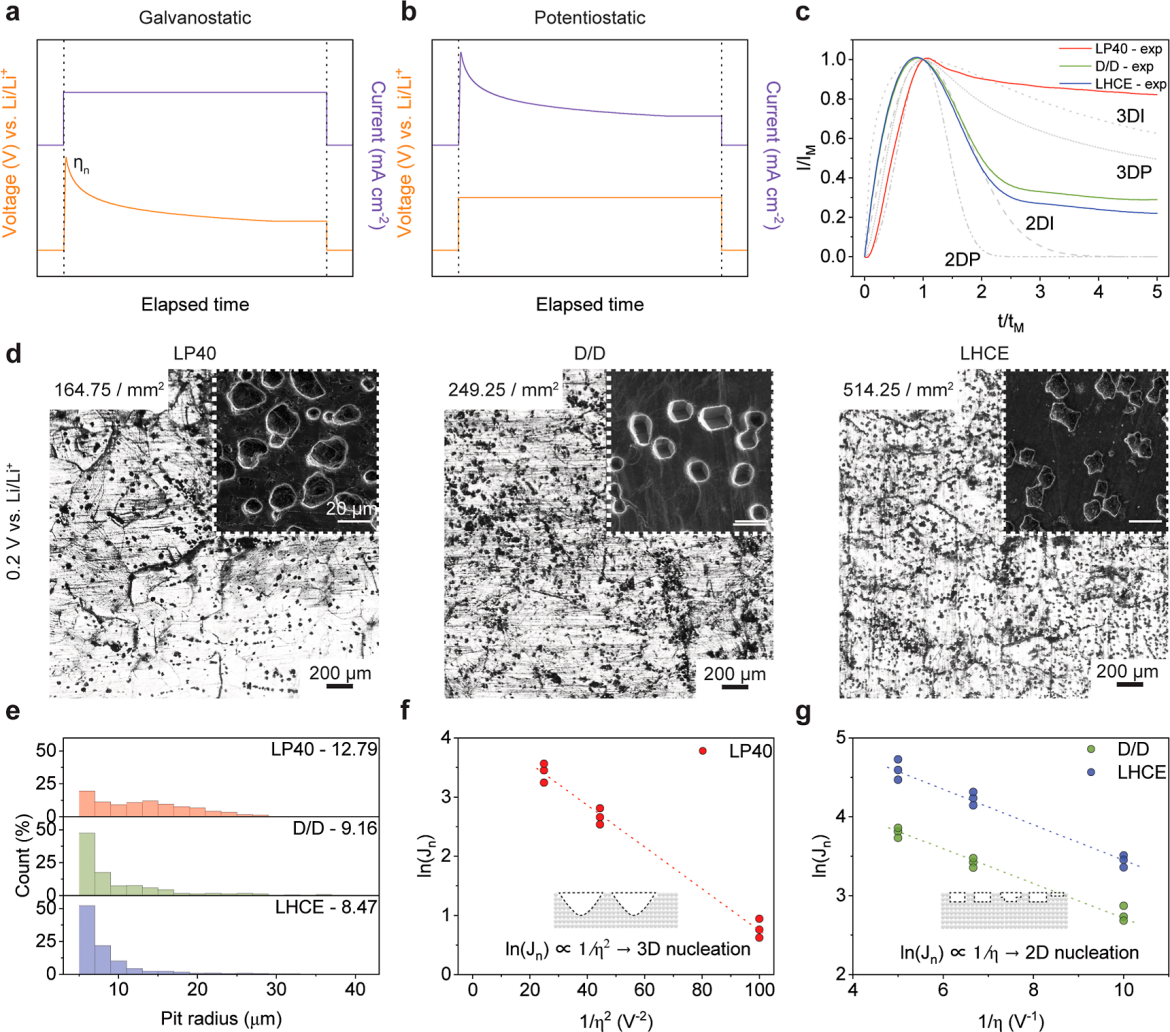
Static nucleation of Li pitting under potentiostatic stripping. Schematic voltage and current profiles under (a) galvanostatic and (b) potentiostatic stripping. (c) Dimensionless current–time transients stripped at 0.2 V in comparison with the classical 2D and 3D nucleation models. “I” denotes instantaneous, and “P” denotes progressive nucleation. Experimental data (exp) are compared with theoretical nucleation models (dashed/dotted lines), including 2D progressive (2DP), 2D instantaneous (2DI), 3D progressive (3DP), and 3D instantaneous (3DI) nucleation. (d) The morphology of the Li pits after the potentiostatic stripping in LP40, D/D, and LHCE-M47 electrolytes. Insets show the SEM images of Li pits. (e) The radius distribution of pits from (d) and the median pit radius in 3 electrolytes. The correlation of the (f) natural logarithm of nucleation rate (*J*_n_) and the inverse of overpotential square (1/η^2^) and (g) natural logarithm of nucleation rate (*J*_n_) and the inverse of overpotential (1/η). The potentiostatic stripping is at 0.05, 0.1, 0.15, and 0.2 V vs Li/Li^+^ for 0.2 mAh cm^–2^ with a 3-electrode Swagelok cell.

Figure 3c compares the normalized current–time transients of LP40, D/D, and LHCE-M47 to 4 classical nucleation models. The Li is stripped at 0.2 V vs Li/Li^+^. The D/D and LHCE-M47 electrolytes exhibit similar mathematical behaviors that resemble the 2DI model, indicating restricted lateral nucleation and faster current attenuation. This explains the larger *r*/*d* ratio at the early stage of stripping. In contrast, LP40 shows consistency with the 3DI model, reflecting instantaneous nucleation with a hemispherical shape. It should also be noted that for *t*/*t*_M_ > 3, the deviation from the model curves arises from the growth process, where the measured current no longer represents nucleation alone but also contributions from pit expansion.

We selected 4 voltages for potentiostatic measurement: (1) 0.05 V (stripping without pitting); (2) 0.1 and 0.15 V (mixed mode); and (3) 0.2 V (pitting dominant mode), and the stripping capacity is fixed at 0.2 mAh cm^–2^. When stripped at 0.05 V vs Li/Li^+^ (Figure S8), there is no pitting formation as Li is stripped along certain grains and grain boundaries.^8^ In Figure S9, when the stripping voltage increases to 0.1 V, the pitting forms, and the pitting density is 55, 132.75, and 186.25 per mm^2^ for LP40, D/D, and LHCE-M47, respectively. When it further increases to 0.2 V, the pitting density increases to 164.75, 249.25, and 514.25 pits per mm^2^, as exhibited in Figure 3d. Figure 3e shows the pit radius distribution when stripped under 0.2 V for 0.2 mAh cm^–2^, where the median pit radius is 12.79, 9.16, and 8.47 μm for LP40, D/D, and LHCE-M47, respectively. The pit is the smallest formed in LHCE-M47. The pitting density follows this trend: LHCE-M47 > D/D > LP40, indicating that Li pitting has a larger nucleation rate *J*_n_ in LHCE-M47. Figure 3f shows ln­(*J*_n_) vs 1/η^2^ plot in LP40, where it follows a good linear fitting, confirming the 3D nucleation mode of Li pitting. ln­(*J*_n_) vs 1/η fitting of D/D and LHCE-M47 is shown in Figure 3g, which are consistent with 2D instantaneous nucleation. Under the same stripping overpotential, the Li-metal anode in the LHCE-M47 electrolyte shows the largest nucleation density and the smallest pits. These results suggest that the electrolyte not only determines the nucleation density and pit size but also fundamentally alters the nucleation mode and kinetics. Specifically, differences in the electrolyte composition influence the formation and properties of the SEI, as well as the interfacial charge-transfer resistance and Li^+^ desolvation at the interface.

### The SEI and Charge Transfer in 3 Electrolytes

There are three steps involved during the stripping reaction of Li: (1) the desolvation of Li^+^; (2) the Li^+^ transportation across SEI; and (3) the electron transfer across the interface.^31–33^ All these steps are strongly related to the recipe of the electrolyte.^34,35^ To elucidate the solvation structure and ion speciation behavior in different electrolytes, we performed FT-IR and Raman spectroscopy on LP40, D/D, and LHCE-M47, and the peak assignments are shown in Figure 4a–c. The characteristic vibrational modes were deconvoluted into solvent-separated ion pairs (SSIP), contact ion pairs (CIP), and aggregates (AGG).^36,37^ This enables quantification of the Li^+^ coordination environment. In LP40 (Figure 4a), the FT-IR spectrum is dominated by a sharp SSIP peak, suggesting weak ion pairing and a strong solvation structure with the integral of the SSIP peak area accounting for approximately 97% of the total species. In contrast, the D/D electrolyte exhibits a pronounced CIP contribution (∼74%), as shown in Figure 4b, indicating an intermediate solvation environment where anions and solvent molecules partially coordinate Li^+^ ions. In Figure 4c, the LHCE-M47 represents a significant shift toward AGG features, consistent with a weakly solvated structure resulting from the high salt-to-solvent ratio. Figure 4d summarizes the trend of the solvation strength: LP40 > D/D > LHCE-M47. It should be noted that such desolvation capabilities are measured from the bulk electrolytes.

**Figure 4:**
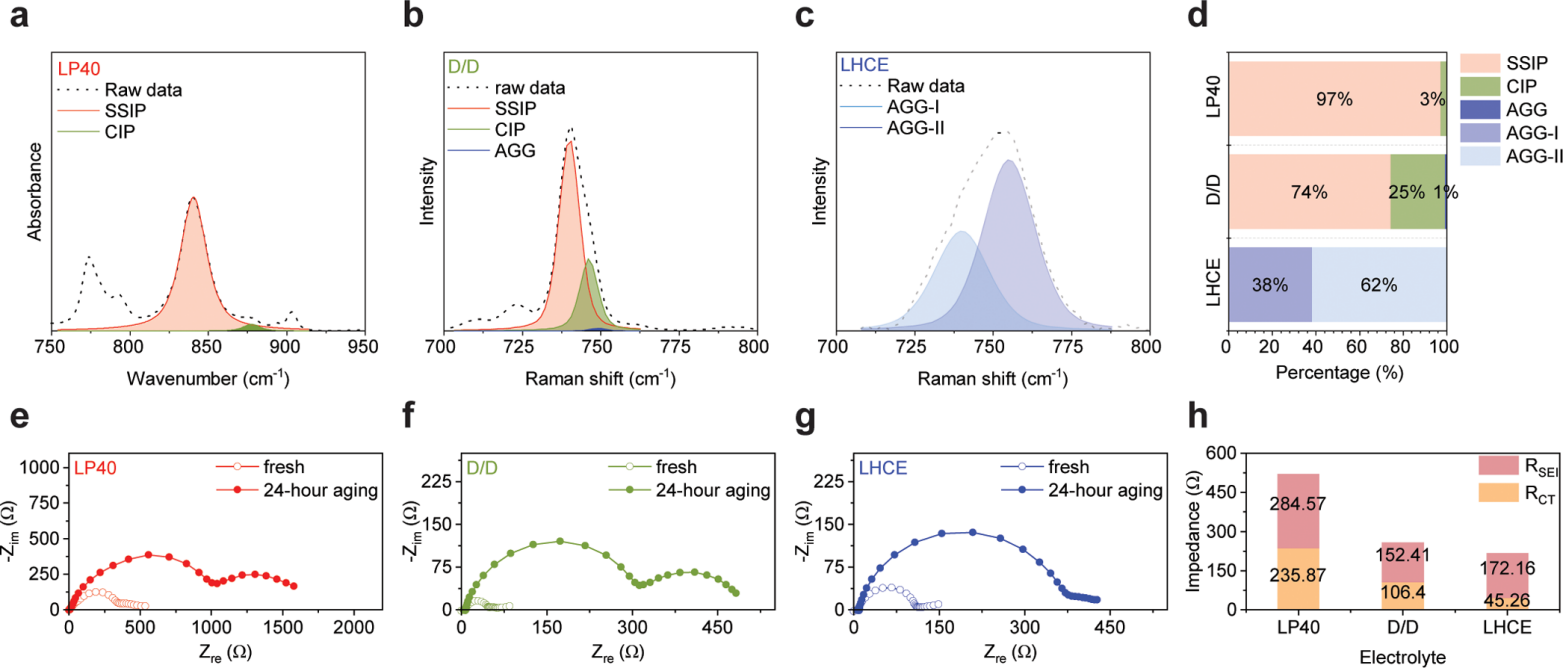
Electrolyte effect on the SEI and charge-transfer resistance. The ion-pairing speciation of (a) FT-IR spectrum for LP40; Raman spectra for (b) D/D and (c) LHCE-M47. The deconvoluted peaks in each panel correspond to SSIP, CIP, and AGG. (d) Proportion of SSIP, CIP, AGG, AGG-I, and AGG-II in different electrolytes. Impedance of Li/Li symmetric cells with (e) LP40, (f) D/D, and (g) LHCE-M47 electrolytes for fresh-assembly and 24 h aging. (h) The SEI and charge-transfer resistance of the Li/Li symmetric cell after 24 h aging.

To understand the interface effect, we assembled Li/Li symmetric cells with different electrolytes and collected EIS spectra. As shown in Figure 4e–g, the impedance increases in all 3 electrolytes after 24 h of aging. The two semicircles at high- and middle-frequency regions correspond to the SEI resistance and charge-transfer resistance.^38^ In Figure 4h, we summarize the *R*_SEI_ and *R*_CT_ of Li/Li symmetric cells after aging 24 h with 3 electrolytes. It should be noted that the *R*_SEI_ and *R*_CT_ values represent the combined contributions of both Li anodes. Therefore, the actual *R*_SEI_ and *R*_CT_ values are half of the values from EIS measurements. *R*_SEI_ in LP40 begins at 158.35 Ω and then slowly increases to 284.57 Ω after 24 h. Similarly, the charge-transfer resistance *R*_CT_ starts at 104.97 Ω and eventually reaches 235.87 Ω after 24 h. For D/D electrolytes, *R*_SEI_ and *R*_CT_ begin with 16.00 and 21.68 Ω, increasing to 152.41 Ω and 106.42 Ω after 24 h aging. The *R*_SEI_ and *R*_CT_ in LHCE-M47 electrolyte initiate from 98.83 and 15.24 Ω, then reach 172.16 and 45.26 Ω after 24 h of aging. Thus, the SEI formed in LP40 has the highest SEI and CT resistance, which explains its superior passivity in Figure 1g. This highlights a trade-off between interfacial kinetics and passivation. For ether-based electrolytes D/D and LHCE-M47, their interfaces have lower *R*_CT_ and *R*_SEI_ than LP40 at the initial stages and final stages.

### SEI and Interface Effect on Nucleation of Pitting

As revealed in Figure 1g, the onset potentials of Li pitting are similar (∼0.1 V vs Li/Li^+^) for LP40, D/D, and LHCE-M47 electrolytes. Under galvanostatic conditions, SEI with a larger resistance leads to a higher overpotential on the Li-metal anode. Figure S10 shows the correlation (*R* ^2^) between the overpotential and *R*_SEI_/*R*_CT_/*R*_SEI_ + *R*_CT_ under galvanostatic stripping. The *R*_SEI_ exhibits the strongest correlation with overpotential across all current densities, indicating that SEI resistance governs overpotential during galvanostatic stripping. Additionally, Figure S11 shows that in the LP40 electrolyte, Li pits exhibit a more faceted morphology without the formation of SEI. In the absence of an SEI, the anisotropy of Li pitting is primarily governed by substrate effects. As the SEI grows, its influence becomes dominant and ultimately overrides the substrate-driven anisotropy.^5^ Figure S12 summarizes the *R* ^2^ values for ln­(*J*_n_) vs the inverse of *R*_SEI_/*R*_CT_/*R*_SEI_ + *R*_CT_ under potentiostatic measurement. The strongest correlation is observed for 1/*R*_CT_, suggesting that the charge-transfer resistance predominantly governs the nucleation rate and nucleation mechanism. This observation is consistent with the nucleation of Li electrodeposition, where the nucleation rate is proportional to the exchange current density.^39^ With 2D nucleation mode, pitting in ether-based electrolytes (D/D and LHCE-M47) shows higher *r*/*d* ratios and generates smaller surface roughness on the Li-metal anode, as shown in Figure S13. Below the stripping current density of 2 mA cm^–2^, 2D nucleation of Li pitting generates less surface roughness and area than 3D nucleation. For electrolyte design, faster charge transport kinetics is desirable, which will lower the surface roughness by homogenizing nucleation of pitting.

## Conclusions

In this study, we explored the SEI and interface effect on the nucleation of pitting on the Li-metal anode. With galvanostatic stripping, we found that the pit size in ether electrolytes is generally larger than that in carbonate electrolytes. This is related to the smaller overpotential of stripping in ether electrolytes. With potentiostatic stripping, we found that ether-based electrolytes (D/D and LHCE-M47) yielded smaller pits and higher nucleation density. By fitting the *i*–*t* curve and analyzing the relationship of nucleation density *J*_n_ and overpotential η_n_, we found that carbonate and ether have different nucleation modes. LP40 has a relatively slower nucleation rate, similar to that of 3D nucleation, while D/D and LHCE exhibit a faster 2D nucleation mode. By analyzing the shape and depth, we found that pits formed in LP40 are predominantly rounded, whereas those formed in D/D and LHCE-M47 exhibit more faceted in-plane morphologies. The morphology of the pit is closely related to the thickness of the SEI. By clarifying the role of the SEI and charge transfer on the nucleation of pitting, we found that charge transfer plays a dominating role in the nucleation speed. With faster interface charge transfer on the Li-metal anode, the nucleation rate and homogeneity of pitting will be greatly promoted, rooted in the 2D nucleation mode. Decoupling the effect of *R*_SEI_, *R*_ct_, and stripping overpotential on nucleation of pitting, this study will inspire more charging profiles and electrolyte recipe design for next-generation Li-metal batteries.

## Methods

### Materials

The lithium foils (Alf 750 μm) are thinned to 150 μm by a TMAXCN roller. The lithium foil is freshly made to assemble the cells, avoiding the formation of a passivation layer from oxidation/aging. 1 M LiPF_6_ in the EC/DMC = 50/50 (v/v) electrolyte, 1,3-dioxolane, 1,2-dimethoxyethane, bis­(trifluoromethane)­sulfonimide (LiTFSI), and LiNO_3_ are purchased from Sigma-Aldrich. 1,1,2,2-tetrafluoroethyl 2,2,3,3-tetrafluoropropyl ether (TTE) is purchased from Synquest Lab. All of the Li salts are dried in a vacuum chamber at 70 °C overnight, and all of the solvents are purified using molecular sieves.

### Preparation of Cells

2032-type coin cells are used for the two-electrode test with lithium foil as the working electrode and copper foil as the counter electrode. Lab-made 150 μm lithium foils with a diameter of 7/16 in. are utilized (total area of 0.97 cm^2^). The separator is Celgard 2325 with a 3-layer structure. The cells are pressed using an MSK-110 hydraulic crimping machine (MTI) under a pressure of 1000 psi. Before the electrochemical stripping, the assembled cells rest for 24 h to ensure a uniform and consistent SEI layer formation. Cells are disassembled by the MSK-110 hydraulic crimping machine with the disassembly module to open the cells and remove the stripped lithium foils. The as-stripped foils are then cleaned with DEC or DME to remove the residual salts. The 3-electrode cells are assembled with a commercially available Swagelok cell, with an electrode diameter of 12 mm. The working electrode is lithium foil, and the counter electrode is copper foil. The reference electrode is either aged lithium foil or half-cycled Li_4_Ti_5_O_12_ (LTO).

### Electrochemical Measurement

The assembled cells are tested by the LANDT battery test system 3200A. The lithium anodes are stripping under the current density of 0.5, 1, 2, 3, 4, and 5 mA cm^–2^ under a constant capacity of 0.2–2 mAh m^–2^. The current density and capacity are carefully chosen as a higher capacity leads to the merging of pits, while a lower capacity makes it challenging to distinguish individual pits under optical microscopy. In our experiments, we measure the stripping overpotential via both 2-electrode (coin cell) and 3-electrode (Swagelok cell) configurations to ensure accurate measurement of the true stripping overpotential. The details can be found in our previous work. The overpotential during galvanostatic stripping is the nucleation overpotential (η_n_). We repeated the galvanostatic stripping under different current densities in 3 electrolytes at least 3 times, and the average overpotential and standard deviation were obtained. The constant voltage stripping and EIS measurements are measured by the AMETEK PMC-1000 series. The constant voltage stripping tests use a 3-electrode Swagelok cell to accurately control the stripping potential from 0.05 to 0.2 V vs Li/Li^+^. The EIS test is conducted with Li/Li symmetric coin cells with a frequency from 1 M Hz to 0.1 Hz.

### Imaging and Analysis

The stripped lithium anode is imaged by the ME580TA-PZ-2L-18M3 optical microscope located inside the glovebox. For pit size and geometry measurements, 10× magnification optical images are used for better resolution. MATLAB’s image processing tools are employed to isolate individual pits from optical microscopy images and measure their radius and circularity. This approach yields a quantity of data sets (ranging from 200 to 1000 pits for a single measurement), enabling unbiased statistical analysis of pit geometry. Detailed procedures are provided in our previous work.^8^ For the CLSM measurement, each stripped lithium foil was placed between a glass slide and a coverslip and then sealed with Kapton tape. The three-dimensional morphology of the resulting Li pits was characterized using a VK-X3100 confocal laser scanning microscope (Keyence). Images were acquired with 20 and 50× objective lenses and subsequently stitched to cover a sufficient area containing a representative number of pits. Data analysis was performed by using VK-X 3000 Multifile Analyzer software (Keyence). The CLSM measurements allow us to quantify the concave volume associated with Li pits (total concave volume) from the stripped Li anode. Because the total stripped volume corresponding to the applied capacity (0.2 mAh cm^–2^) is fixed, we can determine the fraction of Li removed via pitting as the ratio of the total concave volume to the total stripped volume. The remaining fraction (total stripped volume–total concave volume) corresponds to stripping without pitting. The morphology of the samples was also characterized by scanning electron microscopy (Verios, Thermo Fisher Scientific) under an accelerating voltage of 5 kV and a beam current of 0.4 nA.

## Supplementary Material



## Notes

Details regarding the electrolyte selection; critical radius and nucleation rate in CNT; 4 types of classical nucleation models; optical microscopy images of Li pitting in LHCE-M47 and D/D; 2-electrode and 3-electrode overpotential under galvanostatic stripping; ionic conductivity of 3 electrolytes; OM images of pits with increasing stripping capacity; circularity of pits in D/D and LHCE-M47; morphology of Li after potentiostatic stripping; linear regression of overpotential vs *R*_SEI_/*R*_CT_/*R*_SEI_ + *R*_CT_; morphology of Li pits LP40 without SEI formation; linear regression of ln­(*J*_n_) vs 1 over *R*_SEI_/*R*_CT_/*R*_SEI_ + *R*_CT_; and roughness of stripped Li in 3 electrolytes (PDF)
