# Supplementary material for: Solid Electrolyte Interphase and Interface Effect on the Nucleation of Lithium Pitting
Source: ACS Nano. 2026 Jan 17;20(4):3565–73. doi: 10.1021/acsnano.5c16454 (PMC12875022; doi:10.1021/acsnano.5c16454)
Supplement: Supplementary file 1 [file nn5c16454_si_001.pdf]

Supporting Information for

Solid Electrolyte Interphase and Interface Effect on the Nucleation of Lithium Pitting

Hanrui Zhang[1], Weixi Tian[2], Yanjun Guo[1], Dongliang Chen[1], Feifei Shi[1, 2, *]

[1] John and Willie Leone Family Department of Energy and Mineral Engineering, The Pennsylvania State University, University Park, Pennsylvania, 16802, United States.

[2] Department of Materials Science and Engineering, The Pennsylvania State University, University Park, Pennsylvania, 16802, United States.

* Corresponding author

Email address: feifeishi@psu.edu

Note S1 Selection of electrolytes.

Electrolyte selection is crucial for optimizing the performance of lithium-based batteries, with carbonate and ether-based electrolytes being the most extensively studied candidates.[1] LP40 (1M $LiPF_6$ in ethylene carbonate (EC) / diethyl carbonate (DEC)) is a typical carbonate-based electrolyte, and it is widely used in lithium-ion batteries owing to its high ionic conductivity and compatibility with graphite.[2] However, it suffers from poor compatibility with lithium metal anodes due to the formation of lithium dendrites and low coulombic efficiency (CE).[3] In contrast, ether-based electrolytes have superior compatibility with lithium metal, for example, 1M Lithium bis(trifluoromethane)sulfonimide (LiTFSI) in 1,3-dioxolane (DOL) / dimethoxyethane (DME) with 1 wt.% $LiNO_3$ (abbreviated for D/D).[4] The addition of $LiNO_3$ can prevent the shuttle effect of polysulfide in Li-S batteries and extend the cycle life.[5] Unfortunately, most ether-based electrolytes are easily oxidized when they are above 4 V vs. Li/Li^+, making them unsuitable for the 4V-class cathode and hindering the total energy density.[6] In recent years, a series of localized high-concentration electrolytes (LHCE) has been developed to overcome the above issues. By incorporating the super-concentrated electrolytes with a non-solvating solvent (diluent), LHCE can achieve high coulombic efficiency and compatibility with the 4-V class cathode.[7]

Note S2 The critical radius and nucleation rate in classic nucleation theory (CNT).

In CNT, the overpotential (η) serves as the driving force, and it determines the critical radius (r_c) and the nucleation rate (J).[8] In the critical radius of pitting nucleation is governed by the following equation:

$$r_c = \frac{\gamma}{ne\eta} V_a \quad (1)$$

Where γ is the interfacial energy, n is the number of electrons, e is the elementary charge of the electron, V_a is the atomic volume of lithium, and η is the electrochemical overpotential.

For nucleation rate J_n (s^{-1}), it follows:[8, 9]

$$J_n = N_n \mathrm{Zjexp}\left(-\frac{16\pi\gamma^3\Phi(\theta)V_a^2}{3kTn^2e^2\eta^2}\right) \ (3D\ nucleation)\ (2)$$

$$J_n = N_n \mathrm{Zjexp}\left(-\frac{\Omega\gamma^2 b(\theta)}{kTne\eta}\right) \ (2D\ nucleation)\ (3)$$

Where N_n is the number of active nucleation sites, j is the average rate of cluster growth, Z is the Zeldovich factor, k is the Boltzmann constant, T is the temperature, and Φ(θ) is a function describes how the interactions between the of the new phase and the substrate surface influence the nucleus's shape, Ω is the area occupied by one atom on the surface of the cluster, b(θ) is a geometry factor related to the contact angle.

If we take the natural logarithm for equations (2) and (3), they become:

$$\ln(J_n) \propto \left(-\frac{1}{\eta^2}\right) \ (3D\ nucleation)\ (4)$$

$$\ln(J_n) \propto \left(-\frac{1}{\eta}\right) \ (2D\ nucleation)\ (5)$$

Note S3 Four types of classical nucleation models.

Under the potentiostatic measurement, the current–time transients can be normalized with respect to the peak current (I_M) and its corresponding time (t_M), and subsequently compared to 4 classical nucleation models: 3DI, 3DP, 2DI, and 2DP. The Scharifker–Hills (SH) models (3DI and 3DP) describe planar diffusion-controlled three-dimensional growth of hemispherical nuclei.[10] In contrast, the Bewick–Fleischman–Thirsk (BFT) models (2DI and 2DP) represent two-dimensional lateral growth of cylindrical nuclei.[11] The mathematical expressions of these models are:

$$\left(\frac{I}{I_M}\right)^2 = \frac{1.9542}{\left(\frac{t}{t_M}\right)}\left\{1-\exp\left[-1.2564\left(\frac{t}{t_M}\right)\right]\right\}^2 \quad (3DI)$$

$$\left(\frac{I}{I_M}\right)^2 = \frac{1.2554}{\left(\frac{t}{t_M}\right)}\left\{1-\exp\left[-2.3367\left(\frac{t}{t_M}\right)^2\right]\right\}^2 \quad (3DP)$$

$$\frac{I}{I_M} = \frac{t}{t_M}\exp\left[0.5-0.5\left(\frac{t}{t_M}\right)^2\right] \quad (2DI)$$

$$\frac{I}{I_M} = \left(\frac{t}{t_M}\right)^2\exp\left[0.667-0.667\left(\frac{t}{t_M}\right)^3\right] \quad (2DP)$$

The letter "I" denotes instantaneous, and "P" denotes progressive nucleation. Instantaneous nucleation means all active nucleation sites are activated at the very beginning (t close to 0), the current rises quickly to a peak and then decays rapidly. Progressive nucleation is that nucleation sites are activated continuously over time, with the broader and more sustained current transient.

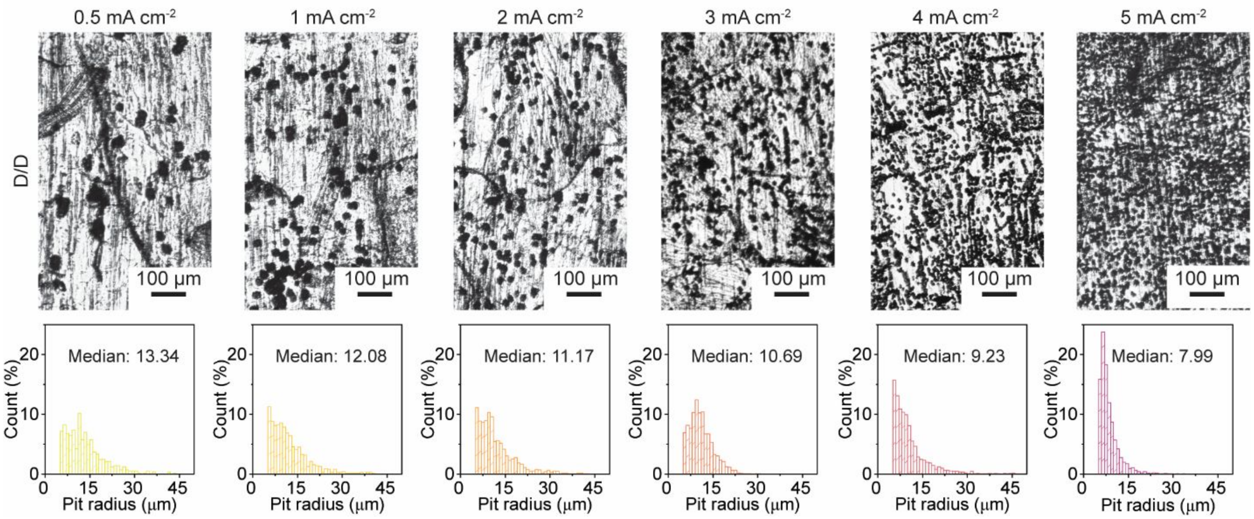

Figure S1 The morphology and pit size distribution of Li after stripping in the D/D electrolyte. The stripping current densities are 0.5, 1, 2, 3, 4, and 5 mA cm^{-2}, respectively. The stripped volume is 0.2 mAh cm^{-2}.

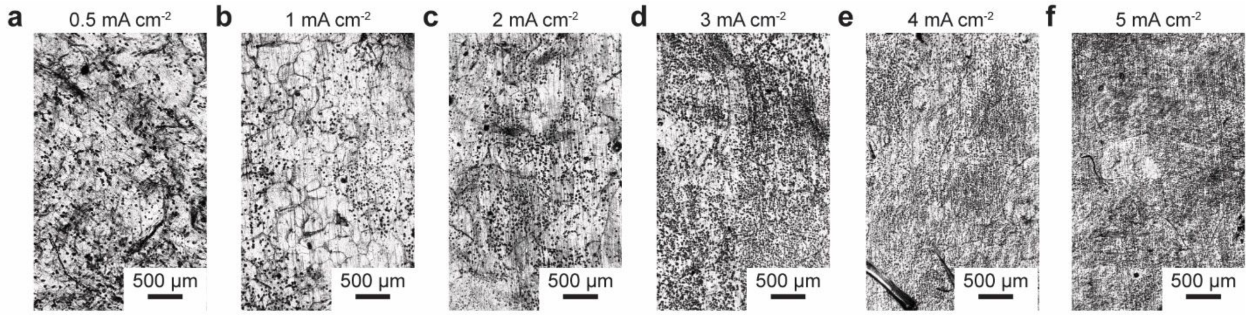

Figure S2 Pit size on a Li-metal anode with various stripping current densities (mA cm^{-2}): (a) 0.5; (b) 1; (c) 2; (d) 3; (e) 4; (f) 5. The electrolyte is LHCE-M47. The stripping capacity is fixed at 0.2 mAh cm^{-2}. Each condition is repeated over 5 times and the images are collected from the samples with overpotential falling in the middle range. The total area of the top row is 10 mm^2.

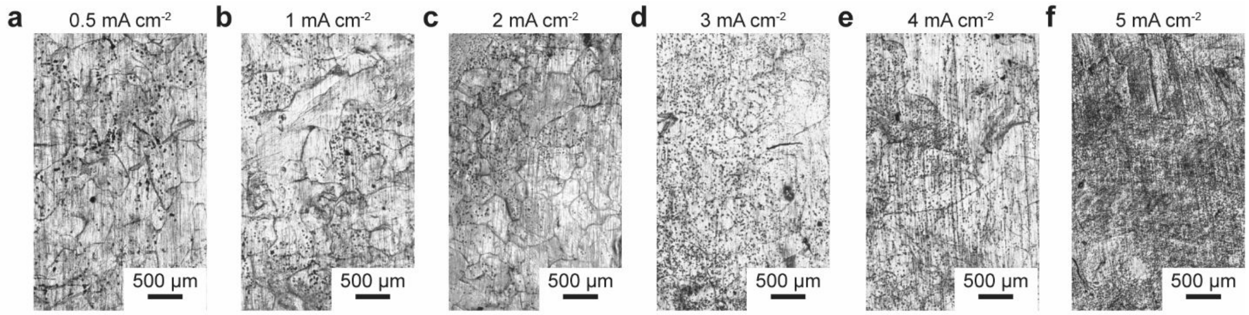

Figure S3 Pit size on a Li-metal anode with various stripping current densities (mA cm^{-2}): (a) 0.5; (b) 1; (c) 2; (d) 3; (e) 4; (f) 5. The electrolyte is D/D. The stripping capacity is fixed at 0.2 mAh cm^{-2}. Each condition is repeated over 5 times and the images are collected from the samples with overpotential falling in the middle range. The total area of the top row is 10 mm^2.

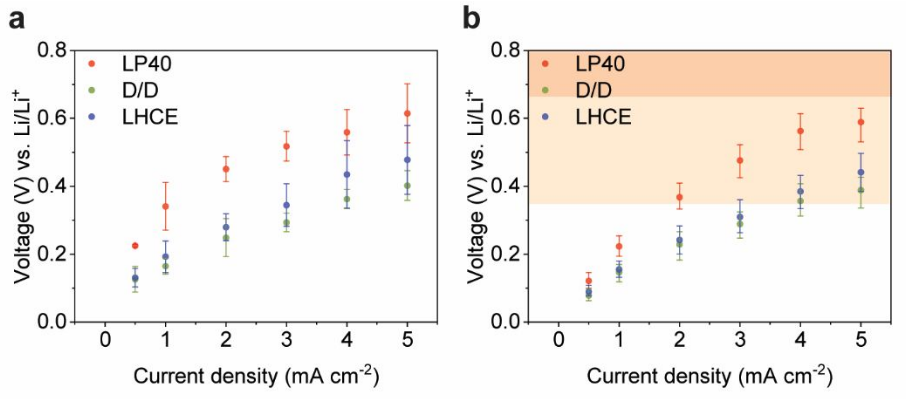

Figure S4 Galvanostatic stripping overpotential for lithium pitting. The stripping overpotential measured by (a) 2-electrode 2032 coin cells and (b) 3-electrode Swagelok cell. Each condition is repeated at least 4 times. The stripping overpotential of LP40 is reproduced from Ref[12]. The orange region refers to the breakdown of ether-based SEI, and the red region refers to the breakdown of carbonate-based SEI.

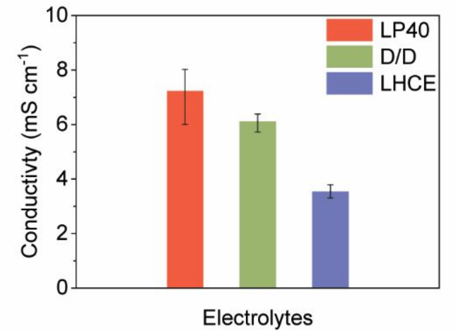

Figure S5 Ionic conductivity of 3 electrolytes. The electrolyte's ionic conductivity is measured by stainless steel symmetric coin cells. (a) LP40; (b) D/D; (c) LHCE-M47.

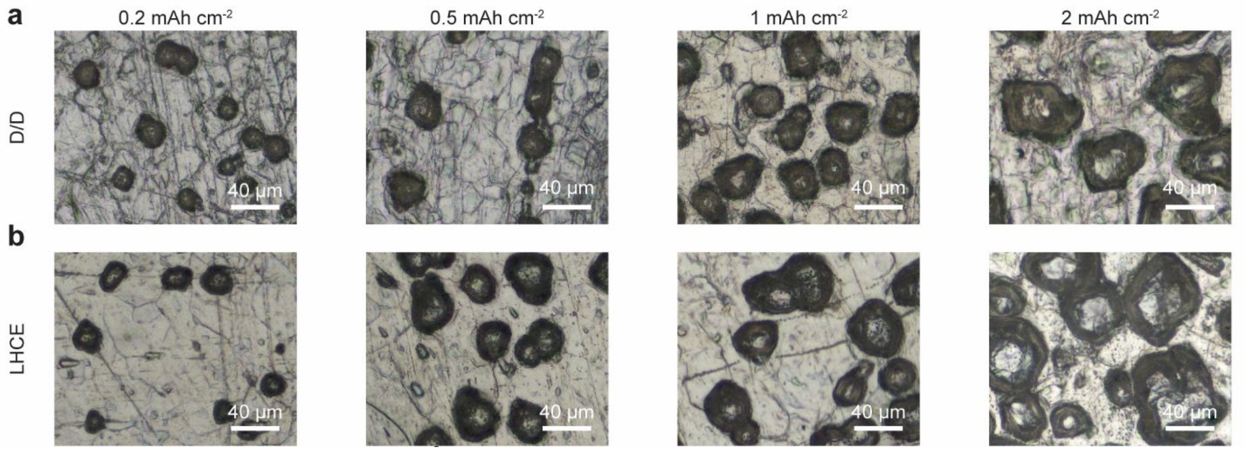

Figure S6 The morphology of the lithium pits after stripping in (a) D/D and (b) LHCE-M47 electrolytes. The stripping current is fixed at 1 mA cm^{-2}. The images are collected from the samples with an overpotential falling in the middle range.

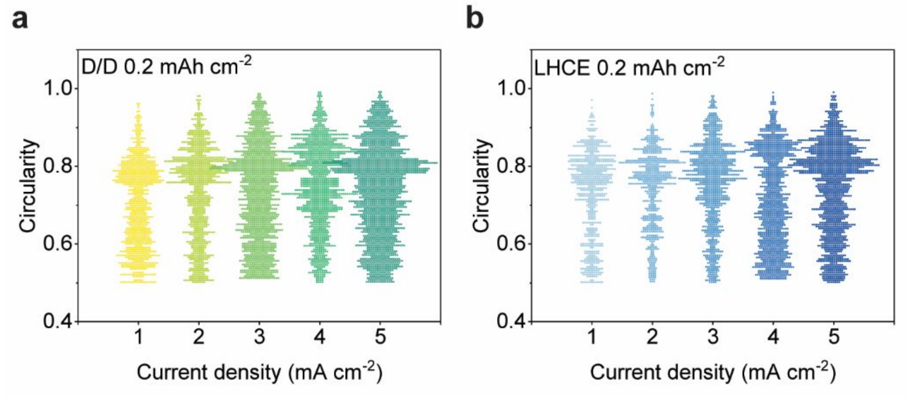

Figure S7 Circularity distributions in different current densities. Circularity of stripped pits in (a) D/D and (b) LHCE-M47. The stripped current is fixed at 1 mA cm^{-2}, and over 500 pits are counted for each capacity.

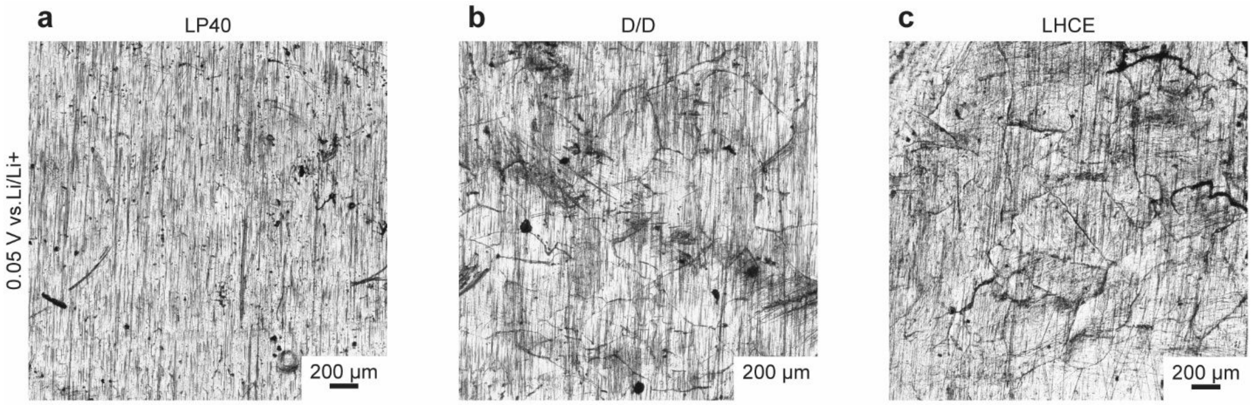

Figure S8 Constant voltage stripping below the onset of pitting. The constant voltage stripping under 0.05 V vs. Li/Li^+ in (a) LP40; (b) D/D; and (c) LHCE-M47 electrolyte. The dark features pre-existing surface imperfections (e.g., surface passivation layers, small concavities, dust/contaminants).

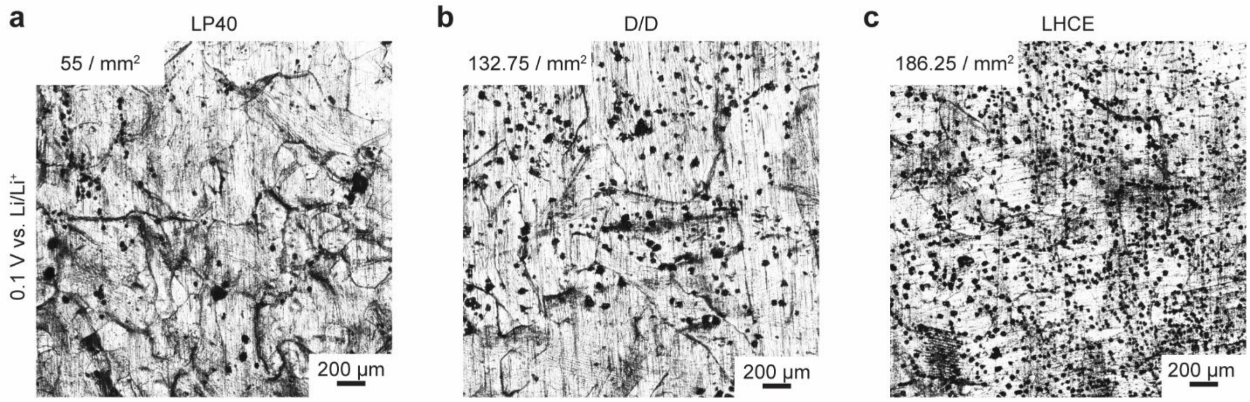

Figure S9 Constant voltage stripping below the onset of pitting. The constant voltage stripping under 0.1 V vs. Li/Li^+ in (a) LP40; (b) D/D; and (c) LHCE-M47 electrolyte.

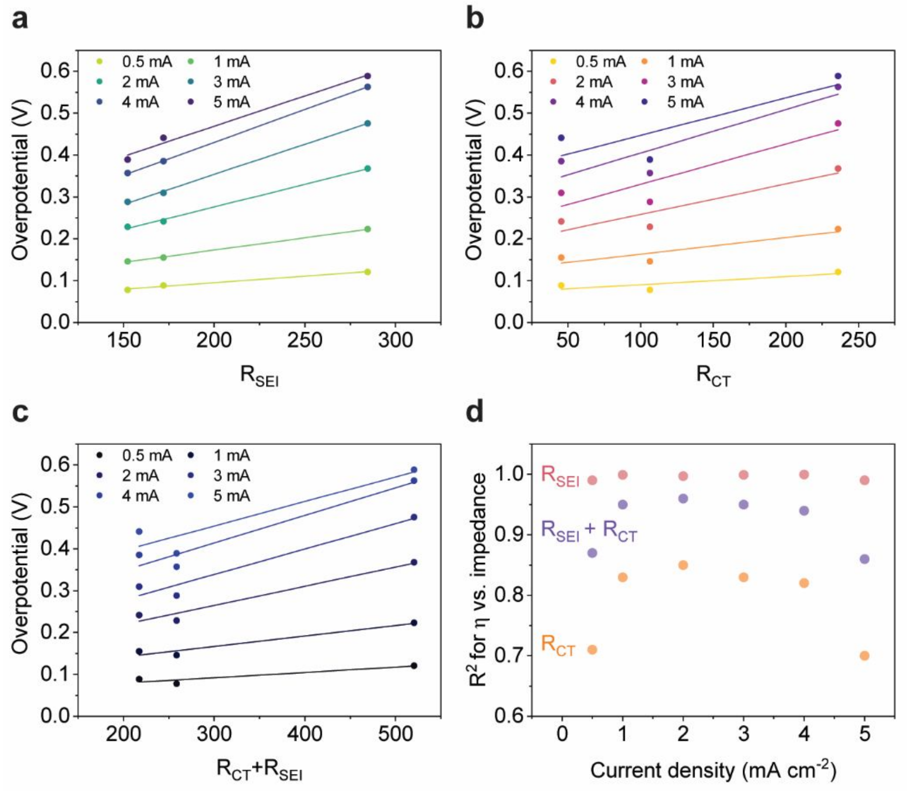

Figure S10 Coefficient of determination R^2 for overpotential vs. $R_{SEI}/R_{CT}/R_{SEI}+R_{CT}$ under galvanostatic stripping.

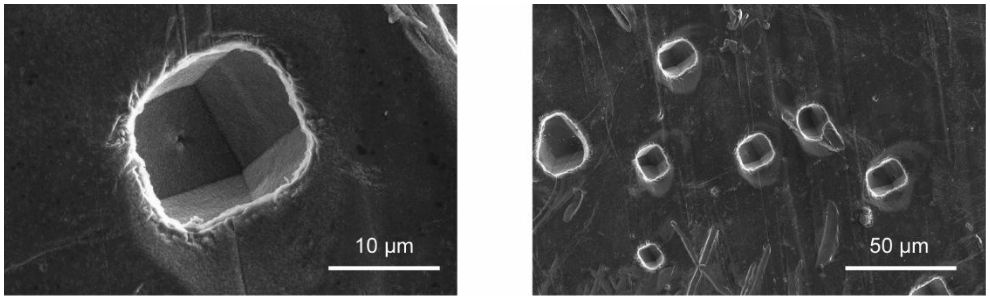

Figure S11 The morphology of Li pits stripped in LP40 (no SEI). The electrochemical stripping tests were performed immediately after cell assembly to minimize SEI growth, and the Li metal was then rapidly transferred to the SEM for morphological characterization. In the absence of an SEI, Li pits formed in LP40 also exhibit an anisotropic growth mode, which is similar to ether-based electrolytes.

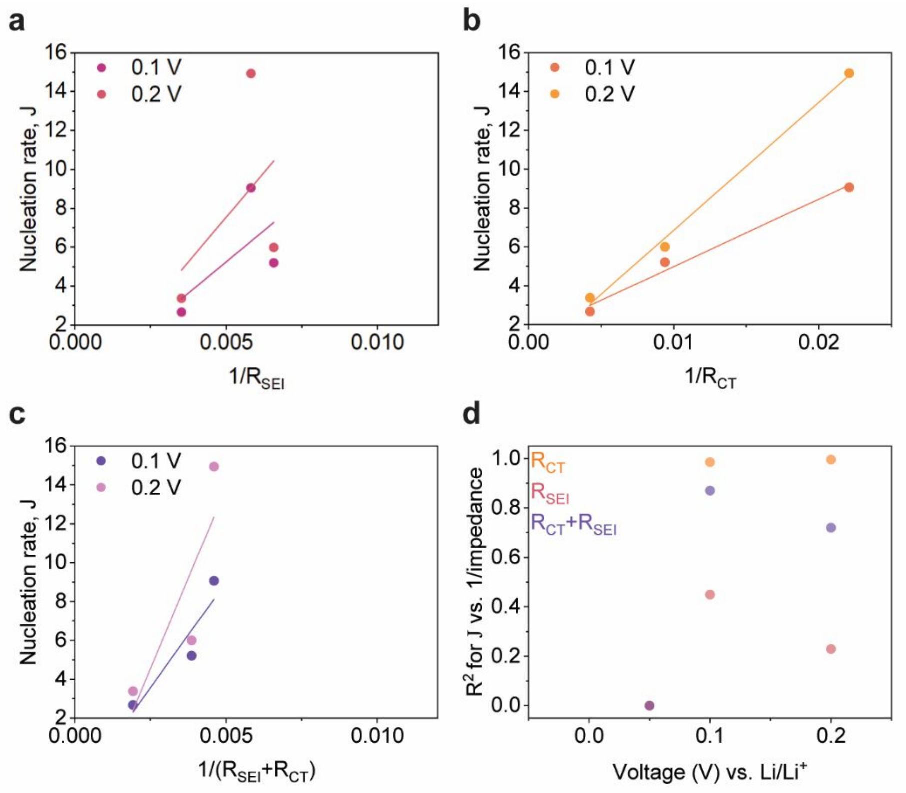

Figure S12 Coefficient of determination R^2 for $\ln(J_n)$ vs. 1 over $R_{SEI}/R_{CT}/R_{SEI}+R_{CT}$ under potentiostatic stripping.

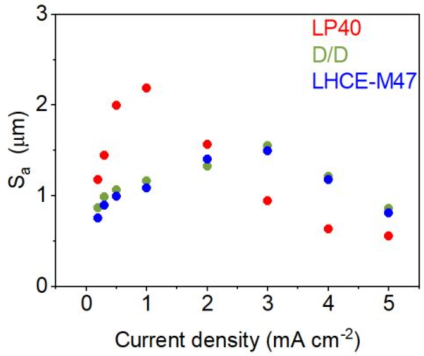

Figure S13 Evolution of the arithmetical mean height (S_a) under different current densities and electrolytes, under a stripped capacity of 0.2 mAh cm^{-2}. S_a is calculated by averaging the absolute values of the surface height deviations from the average plane.

Reference

(1) Xu, K. Electrolytes and Interphases in Li-Ion Batteries and Beyond. *Chemical Reviews* **2014**, *114* (23), 11503-11618. DOI: 10.1021/cr500003w.

(2) Aurbach, D.; Markovsky, B.; Shechter, A.; Ein-Eli, Y.; Cohen, H. A Comparative Study of Synthetic Graphite and Li Electrodes in Electrolyte Solutions Based on Ethylene Carbonate-Dimethyl Carbonate Mixtures. *Journal of The Electrochemical Society* **1996**, *143* (12), 3809. DOI: 10.1149/1.1837300.

(3) Xiao, J.; Li, Q.; Bi, Y.; Cai, M.; Dunn, B.; Glossmann, T.; Liu, J.; Osaka, T.; Sugiura, R.; Wu, B.; et al. Understanding and applying coulombic efficiency in lithium metal batteries. *Nature Energy* **2020**, *5* (8), 561-568. DOI: 10.1038/s41560-020-0648-z.

(4) Liu, Q.; Cresce, A.; Schroeder, M.; Xu, K.; Mu, D.; Wu, B.; Shi, L.; Wu, F. Insight on lithium metal anode interphasial chemistry: Reduction mechanism of cyclic ether solvent and SEI film formation. *Energy Storage Materials* **2019**, *17*, 366-373. DOI: 10.1016/j.ensm.2018.09.024.

(5) Zhang, S. S. Effect of Discharge Cutoff Voltage on Reversibility of Lithium/Sulfur Batteries with LiNO3-Contained Electrolyte. *Journal of The Electrochemical Society* **2012**, *159* (7), A920. DOI: 10.1149/2.002207jes.

(6) Chai, D.; Yan, H.; Wang, X.; Li, X.; Fu, Y. Retuning Solvating Ability of Ether Solvent by Anion Chemistry toward 4.5 V Class Li Metal Battery. *Advanced Functional Materials* **2023**, *34* (8). DOI: 10.1002/adfm.202310516.

(7) Cao, X.; Jia, H.; Xu, W.; Zhang, J.-G. Review—Localized High-Concentration Electrolytes for Lithium Batteries. *Journal of The Electrochemical Society* **2021**, *168* (1). DOI: 10.1149/1945-7111/abd60e.

(8) Bard, A. J.; Faulkner, L. R.; White, H. S. *Electrochemical methods: fundamentals and applications*; John Wiley & Sons, 2022.

(9) Budevski, E. B.; Staikov, G. T.; Lorenz, W. J. *Electrochemical phase formation and growth: an introduction to the initial stages of metal deposition*; John Wiley & Sons, 2008.

(10) Scharifker, B.; Hills, G. Theoretical and experimental studies of multiple nucleation. *Electrochimica acta* **1983**, *28* (7), 879-889.

(11) Bewick, A.; Fleischmann, M.; Thirsk, H. R. Kinetics of the electrocrystallization of thin films of calomel. *Transactions of the Faraday Society* **1962**, *58* (0), 2200-2216, 10.1039/TF9625802200. DOI: 10.1039/TF9625802200.

(12) Zhang, H.; Ulusel, M.; Shi, F. Nucleation of Pitting and Evolution of Stripping on Lithium-Metal Anodes. *ACS Applied Materials & Interfaces* **2024**, *16* (49), 66971-66980. DOI: 10.1021/acsami.4c01530.
